# A Neural Dynamic Architecture for Reaching and Grasping Integrates Perception and Movement Generation and Enables On-Line Updating

**DOI:** 10.3389/fnbot.2017.00009

**Published:** 2017-03-02

**Authors:** Guido Knips, Stephan K. U. Zibner, Hendrik Reimann, Gregor Schöner

**Affiliations:** ^1^Institute for Neural Computation, Ruhr-University BochumBochum, Germany; ^2^Department of Kinesiology, Temple UniversityPhiladelphia, PA, USA

**Keywords:** neural dynamics, dynamic field theory, autonomous reaching, autonomous grasping, online updating

## Abstract

Reaching for objects and grasping them is a fundamental skill for any autonomous robot that interacts with its environment. Although this skill seems trivial to adults, who effortlessly pick up even objects they have never seen before, it is hard for other animals, for human infants, and for most autonomous robots. Any time during movement preparation and execution, human reaching movement are updated if the visual scene changes (with a delay of about 100 ms). The capability for online updating highlights how tightly perception, movement planning, and movement generation are integrated in humans. Here, we report on an effort to reproduce this tight integration in a neural dynamic process model of reaching and grasping that covers the complete path from visual perception to movement generation within a unified modeling framework, Dynamic Field Theory. All requisite processes are realized as time-continuous dynamical systems that model the evolution in time of neural population activation. Population level neural processes bring about the attentional selection of objects, the estimation of object shape and pose, and the mapping of pose parameters to suitable movement parameters. Once a target object has been selected, its pose parameters couple into the neural dynamics of movement generation so that changes of pose are propagated through the architecture to update the performed movement online. Implementing the neural architecture on an anthropomorphic robot arm equipped with a Kinect sensor, we evaluate the model by grasping wooden objects. Their size, shape, and pose are estimated from a neural model of scene perception that is based on feature fields. The sequential organization of a reach and grasp act emerges from a sequence of dynamic instabilities within a neural dynamics of behavioral organization, that effectively switches the neural controllers from one phase of the action to the next. Trajectory formation itself is driven by a dynamical systems version of the potential field approach. We highlight the emergent capacity for online updating by showing that a shift or rotation of the object during the reaching phase leads to the online adaptation of the movement plan and successful completion of the grasp.

## 1. Introduction

Object-oriented reaching and grasping in natural settings, a key element of human-robot cooperation, continues to be a challenge for autonomous robots (Herzog et al., 2012). Humans grasp and handle objects fluently, of course, although these are among the harder movement tasks, learned in infancy (Thelen et al., 1996), but with continued development for close to 10 years of life (Schneiberg et al., 2002). Humans easily reach and grasp objects that they see for the first time or that are partially occluded. They may grasp an object after closing their eyes. Anytime during movement preparation or execution, humans may update the motor plan when the object shifts or rotates (Desmurget and Grafton, 2000). This performance entails, in humans, a close coupling among perceptual processes including gaze control, shift of attention, segmentation, recognition, and pose estimation of the object, as well as between perception and motor processes including initiating, coordinating, and terminating reach and grasp movements.

Robotic approaches to grasping (reviewed in Carbone, 2013) have traditionally made strong demands on what perception delivers, often based on object models. Except for visual servoing, those approaches are most appropriate for static situations with well-known objects. In contrast, recent work has employed simpler perceptual processes, that deliver fast estimates of pose and grasp parameters and enable grasping objects that move with a conveyer belt (Cowley et al., 2013). Another recent line of work learns to extract grasp parameters that are linked to probabilistic models that enable generalization beyond the trained poses, and lead to most impressive real time grasping performance (Huang et al., 2013). Related work learns grasp primitives from demonstration (Herzog et al., 2014), from exhaustive simulation (Curtis and Xiao, 2008), from examples of object categories (Madry et al., 2012), or based on tactile feedback (Platt et al., 2006). Explicit modeling of the uncertainty of grasp parameters provides a potential solution (Li et al., 2016).

This paper is based on two hypotheses. First, we think that we may learn from how humans generate reaching and grasping movements. For instance, as a major theme that we address here, we believe that reaching and grasping is possible in humans with much simpler, lower-level perceptual representations than traditionally assumed in autonomous robotics. The perceptual processes engage attention and enable continuous online coupling to the sensory surface. Another example is at the level of control: The nature of actuation through muscles that act as relatively soft, tunable strings makes it possible to grasp without a precise estimate of grasp points. It is enough to set the equilibrium length of muscles in the hand to a posture inside the object and the muscles will then generate grip forces through their peripheral reflex loops (Santello et al., 2016). In this paper, we address the first, but not yet the second idea.

The other hypothesis is, in a sense, the converse. Many of the neural processes underlying human movement that is directed at objects have not yet been comprehensively understood in neuroscience (Andersen and Cui, 2009; Lisman, 2015). This means that neurally based process models do not stand ready to be imported into robotics. But this also means that how the component processes work together in the nervous system needs to be better understood. Integrated models demonstrate reaching and grasping in neurally grounded ways that may make a contribution to understanding neural function.

Our research agenda is thus to build an integrated model of reaching and grasping based on neural process accounts inspired by the human mind. We do this based on the theoretical framework of Dynamic Field Theory (DFT, see Schöner, 2008 for an introduction, Schöner et al., 2015 for a systematic tutorial), a neurally grounded set of concepts that address visual representations, coordinate transforms, attentive selection, working memory, and behavioral organization. To build and implement a complete model of reaching for and grasping novel objects, we propose a neurally inspired computational architecture.

All processes are modeled as neural dynamics, so that the entire architecture is essentially one big dynamical system. The theoretical framework of Dynamic Field Theory (DFT) provides the means to represent information, to perform detection and selection decisions, to model attention, track time varying input, and to store information in working memory. Instabilities of the neural dynamics create the discrete events from time-continuous processes at which processes are initiated and terminated (Sandamirskaya et al., 2013). The neural dynamics interfaces with attractor dynamics that generate movements and control the robotic arm and hand (Reimann et al., 2011). The model builds on earlier work on scene representation (Zibner et al., 2011a), and on the simultaneous recognition of objects and estimation of their pose (Faubel and Schöner, 2009). We show how neural dynamics enable integrating and organizing all component processes, from the perception to the initiation and termination of robotic movements (Richter et al., 2012).

**Figure 1:**
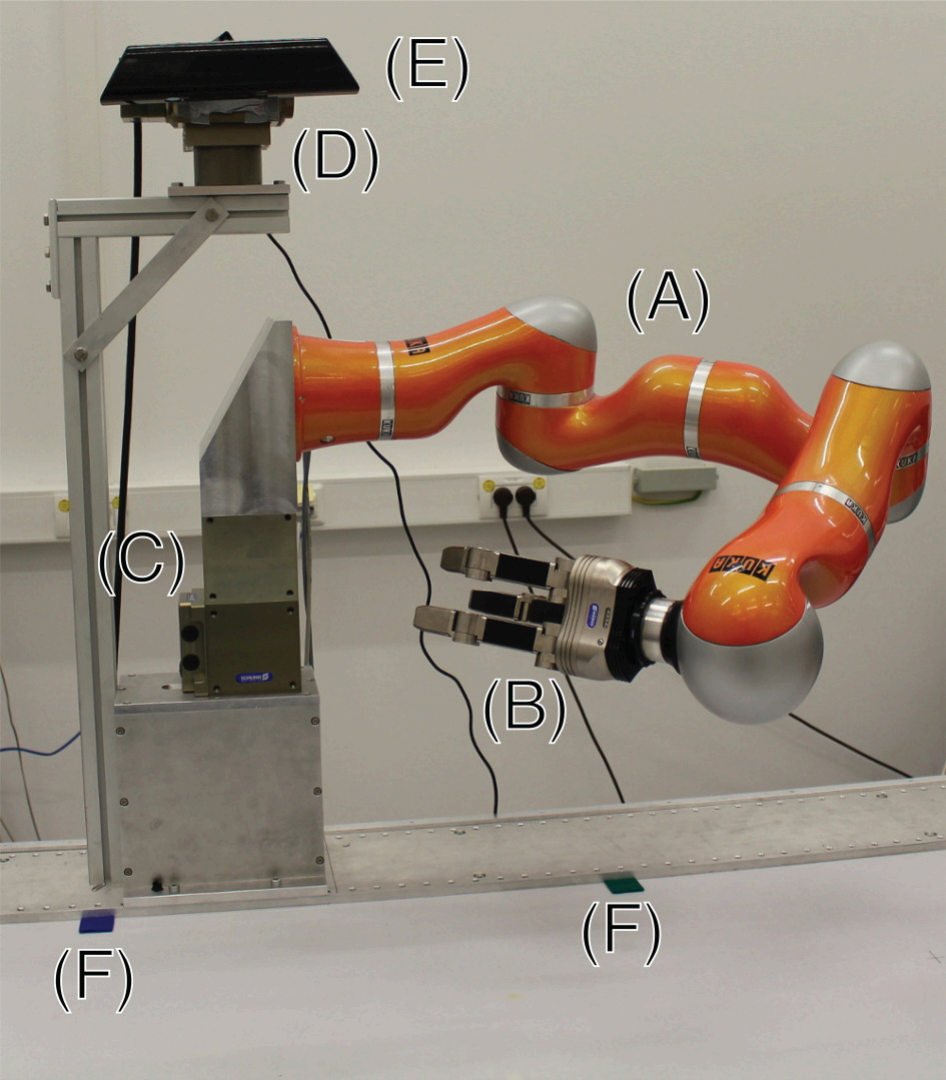
**This figure shows CAREN, the robotic platform used in this work**. It consists of a Kuka LWR4 (A), a Schunk SDH (B), and two Schunk rotary modules, one used as trunk (C), the other as pan-tilt head (D). A Kinect (E) is attached to the head of the robot. Two markers (green and blue) are placed on the table surface (F). They denote the *x*-axis of the table's coordinate system.

The approach is tested on a robotic agent called CAREN consisting of a Kuka LWR4 with seven degrees of freedom, with an attached Schunk Dextrous Hand (SDH) featuring additional seven degrees of freedom and tactile sensors. The arm is mounted on a Schunk PR 90 rotary module with one degree of freedom. We are using a Kinect camera to perceive the scene (see Figure 1).

This work is innovative in two different ways. On the one hand, this work is part of a research program in which robotic demonstrations are used to evaluate theoretical models of human cognition and behavior (Adams et al., 2000). Neural dynamics is a theoretical perspective within this program in which process models are formulated that may be linked to real sensory and motor systems (Erlhagen and Bicho, 2006). Previously, neural dynamics has been used to demonstrate reaching (Strauss and Heinke, 2012; Fard et al., 2015; Strauss et al., 2015). We expand on this work by including the autonomous sequential organization of the behavior and addressing grasping as well. Ours is one of the first demonstrations that cover the complete path from sensing to acting in a difficult task, that includes attention, recognition, estimation, executive control, movement planning, and control. In this demonstration, we integrate four separate neural dynamics models of component processes for scene representation (Zibner et al., 2011a), object classification with concurrent pose estimation (Faubel and Schöner, 2009), behavioral organization (Richter et al., 2012), and movement generation (Reimann et al., 2011).

On the other hand, in direct comparison to approaches to grasping that are unconstrained by analogies with human cognition, the strength of the present work is the capacity to accomodate online updating to changing sensory information, while at the same time addressing the sequential organization of behavior and perception. For instance, work like Huang et al. (2013) has powerful online updating of the grasping action itself, but has a highly simplified perceptual system and limited behavioral flexibility. We think of online updating as a characteristic and attractive property of the neural organization of reaching and grasping and this is why we focus on demonstrating it here.

## 2. Methods

**Figure 2:**
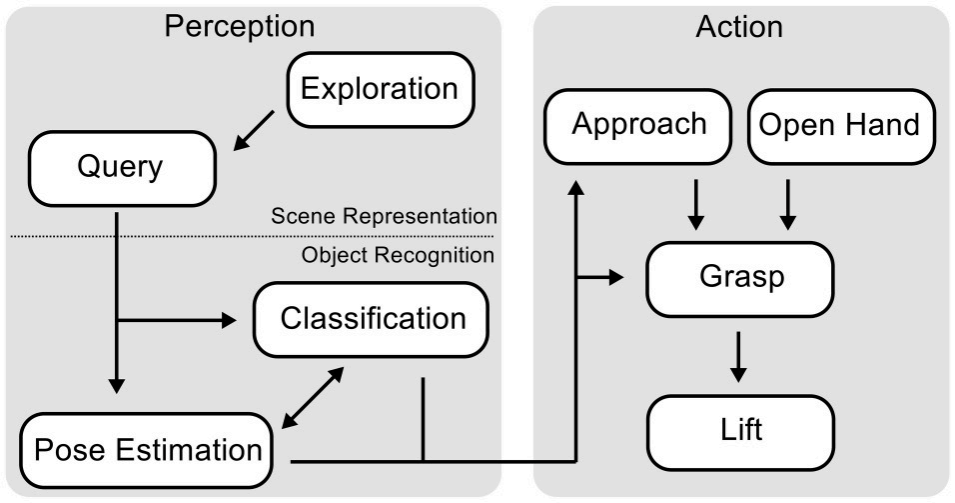
**Schematic overview over the behaviors that make up the reaching and grasping architecture and how they interact**.

We begin by providing a survey over the component processes involved in autonomous grasping and the over-all flow of activation in the neural dynamics architecture (Figure 2). Perception (on the left) consists of scene representation and object recognition. Scene representation entails the processes of visual exploration, which sequentially attends to subregions of the scene that may contain objects and commits an estimate of local height at each attended location to working memory. Visual exploration is a precondition of the query behavior, which processes a cue that defines a target object, brings matching locations into the attentional foreground and thus enables the process of object recognition to take over. Object recognition entails two interacting processes, shape classification, and pose estimation. Shape classification determines the type of grasp that will be used for the current target object, while pose estimation specifies parameters of the reach and the grasp such as hand orientation. Once both processes have converged, a sequence of actions executes the grasp (illustrated on the right). Initially, two behaviors are activated: “Open hand” does what the name suggests and “approach” drives the hand to a point close to the target object while orienting the hand based on a pose estimate. After both behaviors are completed, the “grasp” behavior moves the fingers. Up to that point, online updating of the classification and pose estimation processes is possible, after this point, online updating is suppressed. After detecting contact of the hand on the object's surface through tactile feedback, the “lift” behavior is activated, which raises the arm with the grasped object upwards from the table surface.

Although this description suggests that the individual behaviors and processes are separate modules, in reality they are all just subsets of one large system of differential and integro-differential equations, the neural dynamics, whose solutions evolve continuously in time. These equations are coupled internally according to the architecture and to online sensory inputs. Online updating is thus a pervasive property of the architecture and neural dynamics approaches, in general. We now take a closer look at the elementary building blocks of the architecture to illustrate how neural dynamics and, specifically, DFT, are organize the interaction of the behaviors and processes.

### 2.1. Dynamic neural fields

Dynamic neural fields are the building blocks of Dynamic Field Theory (DFT). Continuous neural activation patterns, *u*(*x, t*), defined over a feature dimension, *x*, evolve in time according to an integro-differential equation that has been proposed as a simplified model of cortical neural dynamics (Amari, 1977):

$$\begin{array}{l} \tau \dot{u}\left(x,t\right)=-u\left(x,t\right)+h+s\left(x,t\right)+\int w\left(x-x\prime \right)\sigma \left(u\left(x\prime ,t\right)\right)dx\prime . \end{array}$$

Here, τ determines the time scale on which activation evolves. The −*u*-term endows this neural dynamics with the fundamental stability mechanism that creates different kinds of attractor solutions under different conditions. The attractor at the resting level, *h* < 0, is stable in the absence of external input, *s*(*x, t*). When such input from other neural fields or from sensory surfaces remains small, the attractor is shifted to *h* + *s*(*x, t*). When inputs become sufficiently strong so that this solution reaches a threshold given by the sigmoidal nonlinearity, σ(·) = 1/(1 + exp(−β·)), this attractor becomes unstable. The system switches to a new attractor state, a localized peak of activation that is sustained by local excitatory and global inhibitory interaction characterized by the interaction kernel, *w*(Δ*x*). The instability at which a switch to such a self-stabilized peak solution occurs is the *detection instability*, used to implement detection decisions in DFT. Localized peaks become unstable at the *reverse detection instability* at lower levels of input. Multi-modal inputs may lead to the formation of a self-stabilized peak at a single location in the field. This is how *selection decisions* are realized in DFT. Under appropriate conditions (for resting level and interaction strength), self-stabilized peaks may remain stable once the inducing localized input, *s*(*x, t*), is removed. Dynamic fields may be analogously defined over multi-dimensional spaces. Such sustained peaks of activation are the model of working memory in DFT. See Schöner et al. (2015) for a systematic exposition of the mathematical and conceptual structure of DFT. The stability regimes described here depend, of course, on parameter values. Typical values of the main parameters of the neural field dynamics used throughout the architecture are: τ = 100 *ms*, β = 100, *h* between −15 and −5, global inhibition between 0.01 and 0.5, excitatory interaction 1, width of exitatory interaction kernel between 3 and 5.

### 2.2. Neural dynamics of behavioral and process organization

Zero-dimensional neural activation fields are essentially discrete activation nodes described by a differential equation analogous to Equation 1:

$$\begin{array}{l} \tau \dot{u}\left(t\right)=-u\left(t\right)+h+s\left(t\right)+w\sigma \left(u\left(t\right)\right) \end{array}$$

This dynamics may have an “off” attractor at negative levels of activation, and an “on” attractor at positive levels of activation. The “off” attractor may disappear in a detection instability at sufficiently high levels of input, *s*. The “on” attractor may disappear in a reverse detection instability at sufficiently low levels of input, *s*. Both attractors may co-exist bistably for intermediate levels of input. Such nodes are used in DFT to represent the activation and deactivation of categories, processes, or behaviors. For the organization of processes and behaviors, pairs of such activation nodes form an executive control unit (ECU, see Richter et al., 2012). When the *intention* node of an ECU is “on,” it provides spatially homogenous excitatory input (a “boost”) to parts of the architecture that is responsible for executing an associated process or behavior. The *Condition of Satisfaction* (CoS) node is activated when sensory or internal inputs are detected that indicate the completion of a process or behavior. CoS nodes inhibit the intention node, turn “off” the associated process or behavior. A third node may be joined to an ECU to represent a working memory of CoS activation, which maintains a record of the past completion of a processing step. Typical values of the parameters of the neural dynamic nodes used throughout the architecture are: τ = 100 *ms*, β = 100, *h* between −1 and −2, global inhibition 0.01.

### 2.3. Visual processing pathway

The autonomous neural dynamics of visual processing controls exploratory attentional processes that build a working memory representation of the scene, which can be queried to activate a particular target object. A second block of processes determines object identity through classification and estimates object pose to determined grasp parameters.

#### 2.3.1. Scene representation

**Figure 3:**
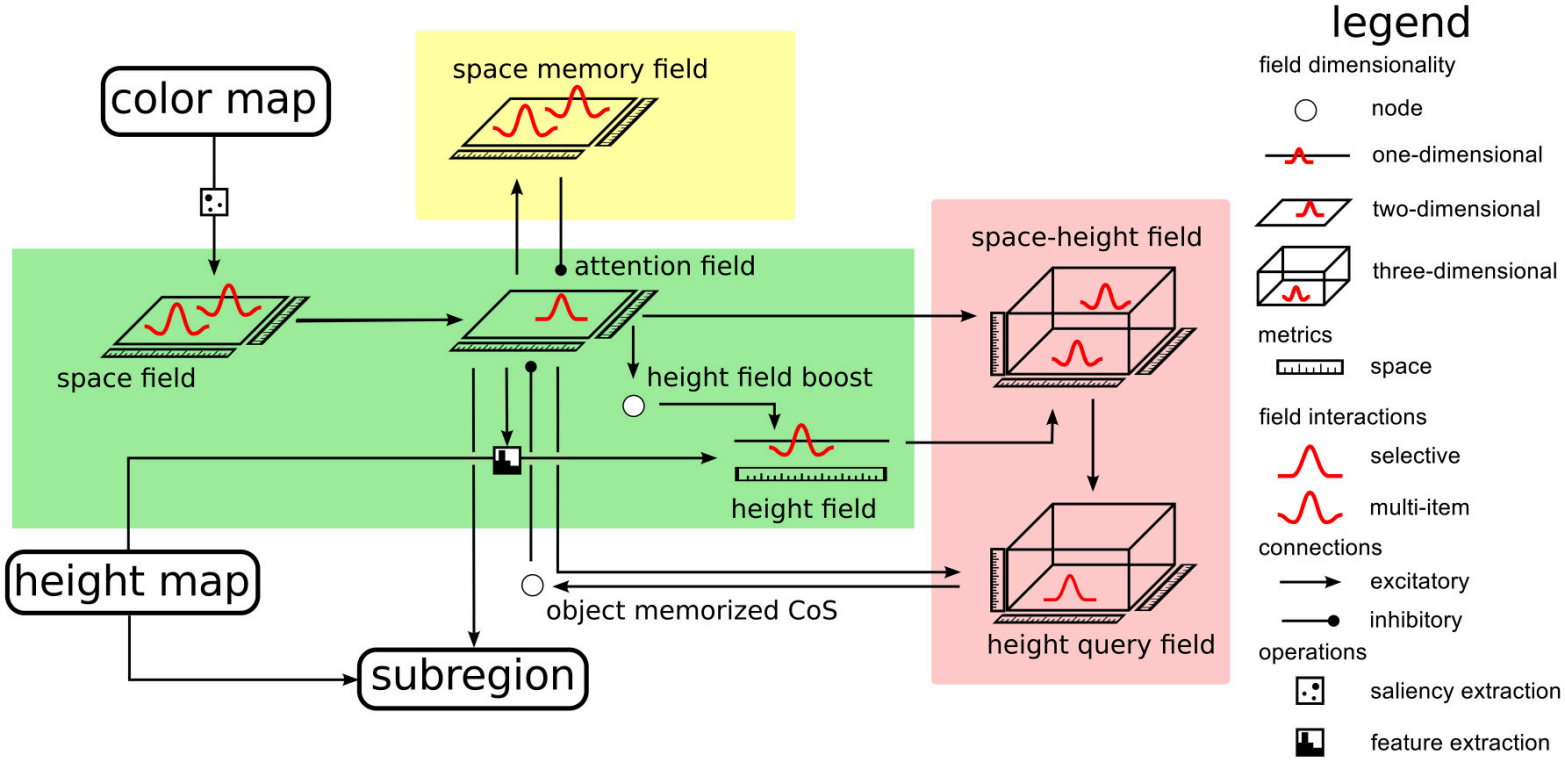
**The portion of the architecture responsible for scene representation**.

The architecture contains an expanded version of a neural dynamic system for scene representation (Zibner et al., 2011a), in which neural dynamic nodes implement a form of process organization (Richter et al., 2012) to enable the autonomous visual exploration of the scene which can transition into a query mode that focusses attention on a target object in the scene. Figure 3 expands this part of the complete architecture. As a cue to locations on a table surface, at which objects may be placed, we use color and visual depth estimates obtained from a Kinect sensor that views the scene in the work space of the robot arm. The idea is that color saturation on the homogeneous table surface guides attention to candidate locations. The height over the table surface estimated at these locations is then used to decide if an object is present (Petsch and Burschka, 2010).

Specifically, we use the Point Cloud Library (Rusu and Cousins, 2011), to find the largest surface in the RGB-D data, which is then identified as the table surface. Height and color maps are extracted in world coordinates. The distribution of saturation in the color map is passed through a sigmoid function and provides input to a neural field defined over the table surface (the space field in the green box of Figure 3). Only regions on the table at which saturation reaches a threshold level drive the neural field through a detection instability and induce a self-stabilized local peak of activation. This effectively suppresses outliers and filters out the noise that is typical of RGB-D data. The field is operated in a dynamic regime in which multiple self-stabilized peaks may coexist. It functions as a salience map for color (Itti et al., 1998).

The color salience space field provides input to a second neural field, the *attention field*, also defined over the table surface. This field is operated in the dynamic regime in which a single localized peak is stable at any time, implementing a selection decision. A self-stabilized peak in this field implements, therefore, selective attention and provides the attentional focus for the rest of the architecture. Height estimates from the subregion on the table, at which activation in the attention field is above threshold, are input into a one-dimensional neural field, the *height field*. The selected spatial region and the neural activation pattern representing height estimates are crossed to provide input into a three-dimensional field, the *space-height field* (on the top right in the red box of Figure 3). For details of how the combination of two lower-dimensional inputs can be used to drive a higher-dimensional field, please refer to Zibner et al. (2011a) or Chapter 9 of Schöner et al. (2015). The space-height field is operated in multi-peak working memory mode, so that it represents the location on the table and height of a potential object as a self-sustained peak, even after the attentional and height inputs are removed. It provides input to a second, three-dimensional field, the *height query field*, that is operated in single-peak mode and thus selects location and the associated height. Input from the attention field controls the location at which input from the space-height field may induce a peak. The height query field thus serves to retrieve a stored object location and height from scene memory.

To guide visual exploration, a multi-peak field over the table surface, the *space memory field*, keeps track of all locations that have come into the attentional focus of the system. A sustained peak of activation is induced each time selective attention is focussed at a location. The space memory field in turns inhibits the attention field and thus biases the process of attentional selection away from locations that have previously been the focus of attention. Autonomous exploration is now organized by a Condition of Satisfaction connection from the height query field into the attention field. Every time a peak has been successfully selected in the height query field, this signals that a memory has been created that matches the currently selected location and currently estimated height. This is the CoS of memory formation and inhibits the attention field, deleting the self-stabilized peak there in reverse detection instability. As a result, the peak in the height query field is no longer supported by selective attention and also decays, releasing the attention field from inhibition. The attention field is ready to select the next location for spatial attention. Inhibitory input from the space memory field now tends to inhibit return to the same location or other recently attended locations, biasing the selection process to new locations with salient color input. This process of visual exploration is continuously ongoing, confirming past memories in the space-height field, updating such memories or creating new such memories as needed.

Autonomous visual exploration can be interrupted at any time by a query for a target object, that triggers the estimation of grasp parameters. The target object can be specified by a spatial cue or by cues of characteristic object features, such as color (for a more detailed description of the querying behavior, see Zibner et al., 2011b). There is a set of neural nodes that activate and deactivate parts of the architecture by boosting or deboosting the resting levels of the associated fields. Not all of those nodes are plotted in the survey over the architecture for simplicity (see a description in the first part of the Results Section for the functional role of these nodes).

#### 2.3.2. Shape classification and pose estimation

Estimation of grasp parameters is based on a recurrent architecture for object recognition (Faubel and Schöner, 2009). In the original work, a weighted sum of object templates, one for each known object, is compared to the current input image. Applying cascaded transformation operations of shift, rotation, and scaling) to the current input and matching the transformed input to each of the memorized templates (by cross-correlation, “C”) yields a competitive weight of each template. Dynamic neural nodes compete with each other, leading to the selection of the template in a classification decisions. In a concurrent process, all templates are weighted with the current activation level of their dynamic neural node and summed. This inverse cascade of image transformations is applied and a match to the input image in each possible pose provides input into neural activation fields defined over the pose parameters for shift, rotation, and scaling. These fields are operated in a single-peak mode so that an emerging self-stabilized peak represents a selection decision among poses. The concurrent upward classification, and downward pose estimation processes converge in closed loop, activating an object identity representation in the set of neural nodes, and a pose estimate in the set of neural fields.

**Figure 4:**
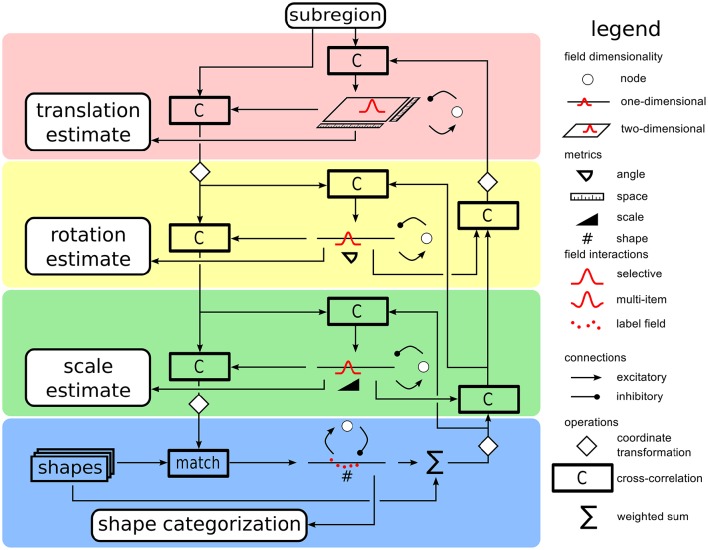
**A sketch of the shape/pose estimation system used to classifify the attended part of the visual scene into a shape category and to concurrently estimate its pose**. Along the downward pathway on the left, the input image is transformed based on the current estimates of translation, rotation, and scaling before being compared to the stored shape templates at the bottom. Along the upward path on the right, the current weighted sum of shape templates is inversely transformed by scaling and rotation operations. Cross-correlations with the input image yield updates to pose estimates. The pose fields in the center column feed into the representation of grasp parameters.

For the present purpose, we replace learned object templates with simple geometric shapes (square, circle, oblong rectangles). The subregion on the table that the attentional focus defines provides the visual input to the shape classification and pose estimation system. The two-layer decision architecture of the original model was further simplified into single layer decision fields connected each to a single inhibitory node that slows down the decision process, allowing multiple candidate peaks to form before a decision emerges. Figure 4 gives an overview of the resulting architecture. The different stages of pose estimation are highlighted by the background color: translation (red), rotation (yellow), and scaling (green). The set of neural nodes that makes shape classification is highlighted in blue.

As the shape classification and poste estimation process converges, it delivers a shape candidate whose location is specification more precisely within the table surface than the attentional systems does. The scaling and rotation estimates together with features of the shape category are used to determined the grasp parameters, represented in the *grasp decision field* Oblong objects with a low height are grasped from above, while cylindrical objects and cuboids with a square base with sufficient height are grasped from the side. The latter objects need different approach movements prior to grasping, since cylinders, unlike cuboids, can be grasped sideways equally well from any direction.

Note, that the estimation process is continuously coupled to visual input through the attentional channel. As a result, changes in the scene are fed into the pose fields enabling online updating of the grasp parameters. In the current version of the model, online updating occurs only with respect to two dimensions of the task, translating, and rotating the gripper.

### 2.4. Reaching and grasping

**Figure 5:**
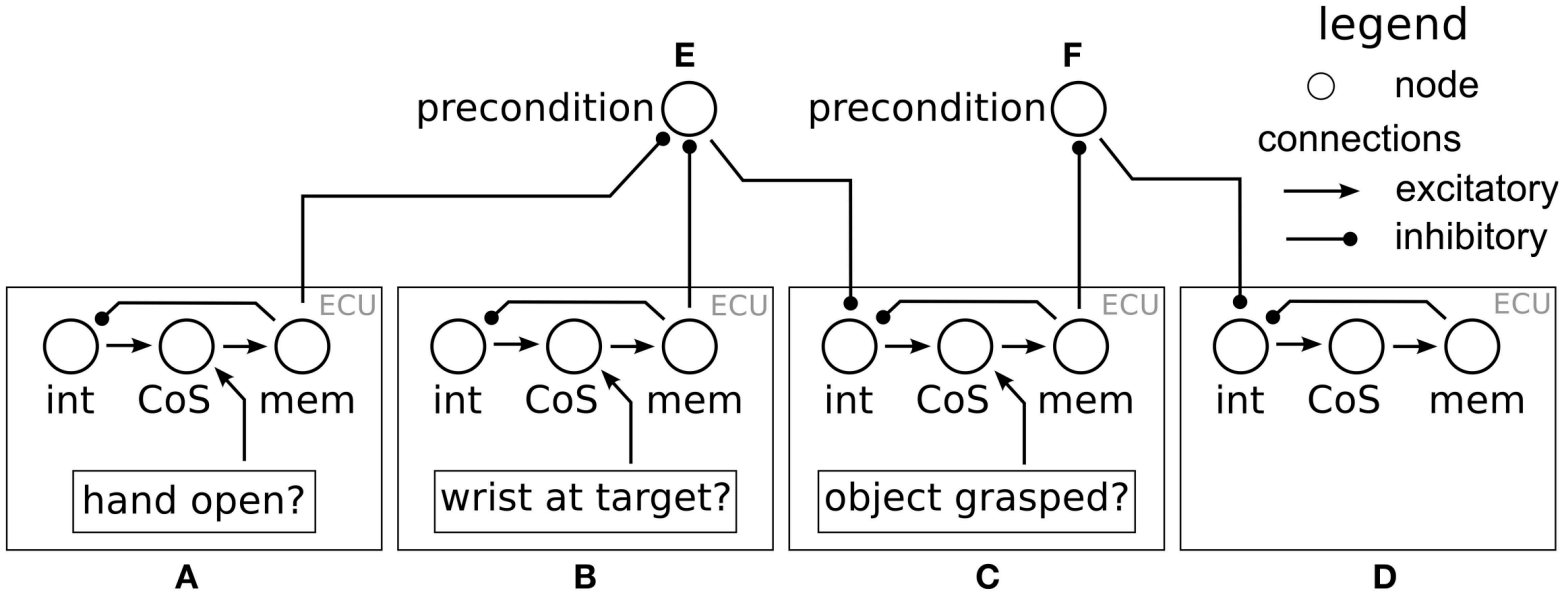
**The neural dynamics of behavioral organization used for movement generation**. There are four ECUs: open hand **(A)**, approach target **(B)**, grasp target **(C)**, and lift **(D)**. The precondition node **(E)** ensures that the grasp behavior is only activated once **(A,B)** have met their CoS. The precondition node **(F)** ensures that the grasp behavior has met its CoS before the object is lifted.

This section explains how data from the scene representation and the shape classification/pose estimation systems are used to generate movement and to grasp an object. The overall scheme is as follows. Depending on the object pose parameters (position, height, rotation, and shape) and the current arm configuration, a desired wrist position and orientation for the hand are computed. These desired values are then set as attractors in a dynamical system that generates movement for the arm. The movement unfolds autonomously in three phases organized by a neural dynamics of the type reviewed earlier (Section 2.2). First, the hand is opened, brought close to the object, and oriented in a way that enables grasping the object. Second, the hand is moved through the remaining distance to the object, and is closed. The third phase begins when the object has been grasped as signaled by the tactile sensors on the fingers. The hand is then moved upward in space, lifting the object. This sequence of actions is generated by a neural dynamics of behavioral organization that is illustrated in Figure 5.

#### 2.4.1. Generating motor commands

Motor commands are generated from desired values for the wrist position and hand orientation using the attractor dynamic approach (Reimann et al., 2011). To move the wrist, movement speed, and direction are controlled separately. The rate of change of movement direction depends on the angle between the current movement velocity, $\vec{v}$, and the vector, $\vec{k}$, from the wrist position to the target position,

(1) $$\begin{array}{l} \phi =\text{arccos }\left(\frac{\left(\vec{v},\vec{k}\right)}{\left|\vec{v}||\vec{k}\right|}\right). \end{array}$$

Reducing this angle to zero corresponds to changing the movement direction into the direction in which the target lies. This constraint is imposed by the dynamics of that angle, given by

(2) $$\begin{array}{l} \dot{\phi }=-\alpha_{\text{dir}}\phi , \end{array}$$

which is linear, simplifying Reimann et al. (2011). Here, α_dir_ is a rate factor.

To translate this constraint into a motion command for the robotic arm, consider the direction, ${\vec{v}}_{⊥}$, in which the movement vector, $\vec{v}$, is changed. It is perpendicular to $\vec{v}$ and lies in the plane spanned by $\vec{v}$ and $\vec{k}$. Computed as:

(3) $$\begin{array}{l} {\vec{v}}_{⊥}=|\vec{v}|\frac{\left(\vec{k}\times \vec{v}\right)\times v}{\left|\left(\vec{k}\times \vec{v}\right)\times v\right|}. \end{array}$$

and normalized to have the same length as $\vec{v}$.

Combining the two equations we determine the direction in which the wrist's velocity vector in cartesian space should change so as to bring the hand closer to the target location:

(4) $$\begin{array}{l} {\vec{f}}_{\text{dir}}={\vec{v}}_{⊥}\left(\dot{\phi }-{\dot{\phi }}_{\text{dev}}\right). \end{array}$$

Here, ${\dot{\phi }}_{\text{dev}}$ is the rate at which the direction from the hand to the target changes due to the movement, $\vec{v}$, of the hand in space. The direction of change lies in the appropriate plane and is proportional to the rate of change of the direction to the target corrected for the rate of change of that direction that is induced by the movement of the wrist in space.

To control movement speed, its rate of change, $\dot{v}$, is proportional to the difference between the current speed, $v=|\vec{v}|$, and a desired speed *v*_des_:

(5) $$\begin{array}{l} {\vec{f}}_{\text{vel}}=\frac{\vec{v}}{v}\left(-\alpha_{\text{vel}}\left(v-v_{\text{des}}\right)\right) \end{array}$$

where α_vel_ is a rate constant. As a contribution to the rate of change of the 3D velocity vector, this contribution lies in the direction of the current velocity.

A third contribution to the dynamics of the hand velocity vector slows down the hand when it is close to the target object in order to reduce any impact in case of misestimation and collision. A local safe control law is proportional to the distance between hand position, $\vec{g}$, and target position, $\vec{p}$:

(6) $$\begin{array}{l} v_{\text{local}}=-\beta_{\text{pos}}\left(\vec{g}-\vec{p}\right), \end{array}$$

and is expanded in vector form as

(7) $$\begin{array}{l} {\vec{f}}_{\text{pos}}=-\alpha_{\text{pos}}\left(\vec{v}-\min\left\{|v_{\text{local}}|,v_{\text{des}}\right\}\frac{v_{\text{local}}}{\left|v_{\text{local}}\right|}\right), \end{array}$$

where α_pos_ and β_pos_ are two rate factors. The introduction of *v*_des_ is a change over the approach of Reimann et al. (2011) intended as a safety measure to delimit movement speeds of the arm.

The rates of change of the hand's velocity vector in Cartesian space are transformed into joint space with the help of the pseudo-inverse, $J_{p}^{+}$, of the Jacobian matrix of the wrist position. The three contributions are then summed after each contribution is weighted with a sigmoidal factor that reflects the distance of the hand to the target. The result is the planned angular acceleration, $\vec{F}$, of the robotic arm in joint space:

(8) $$\begin{array}{l} \vec{F}=\sigma \left(|\vec{k}|-d_{\text{thr}}\right)\left(J_{p}^{+}\cdot \vec{f_{\text{dir}}}+J_{p}^{+}\cdot \vec{f_{\text{vel}}}\right)\\ +\;\left(1-\sigma \left(|\vec{k}|-d_{\text{thr}}\right)\right)J_{p}^{+}\cdot \vec{v_{\text{vel}}}. \end{array}$$

This control strategy for the hand's position largely follows Reimann et al. (2011). The control law of the hand's orientation is formulated for the three Euler angles of the hand used as target angles for the three most distal joints of the arm. The desired rotation matrix *R* can thus be split into three subsequent rotations around three fixed axes. For each of these three most distal joints, θ_*i*_, the angular acceleration ${\ddot{\theta }}_{i}$ is proportional to the deviation between the current joint angle, θ_*i*_, and the desired joint angle, θ_*i*, des_, corrected for by the current angular velocity, *v*_θ_*i*__, induced by the movement of the hand in space according to Equation 9:

(9) $$\begin{array}{l} {\ddot{\theta }}_{i}=-\alpha_{\text{rot}}\left(v_{\theta_{i}}-\beta_{\text{rot}}\left(\theta_{i}-\theta_{i,\text{des}}\right)\right). \end{array}$$

Here, α_rot_ and β_rot_ are rate constants of the dynamics.

Finally, the opening and closing of the hand is controlled through a linear first order dynamical system:

(10) $$\begin{array}{l} \dot{\vec{\theta }}=-\alpha_{\text{hand}}\left(\vec{\theta }-\left(w_{\text{grasp}}{\vec{\theta }}_{\text{closed}}+w_{\text{approach}}{\vec{\theta }}_{\text{open}}\right)\right). \end{array}$$

This dynamical system has attractors either at a joint angle configuration, ${\vec{\theta }}_{\text{open}}$, corresponding to an open hand or at a joint angle configuration, ${\vec{\theta }}_{\text{closed}}$, corresponding to a closed hand. These joint configurations depend on the shape template of the object to be grasped.

#### 2.4.2. Target positions and orientations

Desired positions, *g*, for the wrist are defined for the approach, grasp and lift behaviors, as well as for different grasp types. All approach points for the different object types are updated online. The target point, ${\vec{g}}_{\text{approach}}$, for the approach behavior depends on the grasp type. For vertical objects, it lies in a horizontal plane at two thirds of the object's height at a certain distance from the object that depends on the object's shape. For cylindrical objects, the vector, $\vec{k}$, from the current wrist position to the object position is projected onto the table plane to obtain the direction from which to grasp. For objects with a square base, one of the four sides is selected. This entails computing the inner product of $\vec{k}$ with each of four vectors that are orthogonal to each side. Using four competing neural nodes, the vector that best matches is selected. For objects that are grasped from above, the approach point is at a fixed distance above the object. A weighted sum

(11) $$\begin{array}{l} {\vec{g}}_{\text{approach}}=\frac{1}{n}\underset{i}{\sum }w_{i}{\vec{g}}_{i}, \end{array}$$

over the *n* different object types is used to calculate the instantaneous approach point. The values for *w*_*i*_ are the output values of the *grasp decision field*.

For the target point of the grasping behavior, we use a point on the object vector, $\vec{k}$, at a certain distance, *d*_*i*_, from the object

(12) $$\begin{array}{l} {\vec{g}}_{\text{grasp}}=\frac{1}{n}\underset{i}{\sum }w_{i}d_{i}\frac{-\vec{k}}{\left|\vec{k}\right|}. \end{array}$$

To lift the object, a position, ${\vec{g}}_{\text{lift}}$ is set to a point 50 cm above the table surface located directly above the current position.

The current target position for the movement generation system is then set to the weighted sum over all these different target points

(13) $$\begin{array}{l} \vec{g}=w_{\text{approach}}\;{\vec{g}}_{\text{approach}}+w_{\text{grasp}}\;{\vec{g}}_{\text{grasp}}+w_{\text{lift}}\;{\vec{g}}_{\text{lift}}, \end{array}$$

in which the weight factors are the activation states, *w*_*i*_, of the corresponding behavior.

The orientation of the hand at grasp is chosen so that the opening of the hand points toward the object and the fingers are aligned with the object's surfaces. For tall, narrow objects that are grasped from the side, the palm is chosen to be oriented perpendicular to the table surface. For flat objects that are grasped from above, the palm is oriented parallel to the table. Again a sum is used to obtain the desired orientation of the hand from these contributions, weighted with the activation level of the associated shape class.

## 3. Results

A first goal of our experimental work is to illustrate how the neural dynamic architecture generates the time courses of visual exploration, shape classification and pose estimation, and movement generation. In each case, we aim to show how transitions between different phases of behavior emerge autonomously from the space time continuous dynamical systems. Although we inspect the three components of scene representation, shape classification, and movement generation, one by one, these componets are tightly coupled in the overall neural architecture and evolve in parallel. The second goal is to demonstrate and assess the properties of the neural architecture in achieving reaching and grasping actions. We report three sets of experiments that probe online updating with respect to three dimensions of the task (grasping, translating, rotating). In the following sections, we first give detailed account of the general flow of neural activation through the dynamic fields and nodes. Then we report the results of the three experiments set up to probe specific characteristics of the system.

### 3.1. Time course of scene representation

As long as there is no active cue, the neural architecture of scene representation (Figure 3) performs visual exploration which can be described as follows. The distribution of color over the table surface is captured by the space field that forms one peak at each location with salient color. These peaks provide localized input to the attention field, which generates a single peak and inhibits all alternative locations. This peak masks input from the height map to the height field so that only height measurements within this window contribute. A neural node that detects a peak in the attention field provides a boost to the height field, which together with significant input from the masked height input may induce a peak in this field.

The attention and height fields now contain separate representations of spatial position and height. Spatial input projects as a cylinder localized in space, elongated along height into the three-dimensional space-height field. Height input projects as a slice localized along color, extended along space. Where these inputs intersect, a localized peak arises that binds height to location. This peak induces localized input into the three-dimensional space-height query field, which receives at the same time a cylinder of input localized in space, extended along height from the peak in the attention field. These inputs overlap and create a matching peak in the height query field. A CoS node detects this peak and inhibits the attention field, triggering a cascade of reverse detections in the attention, height, and height query fields, followed by de-activation of the CoS node itself, and a release from inhibition of the attention field. Parallel to this cascade of instabilities, the looking memory field has stabilized a sustained peak at the currently attended location which projects inhibitorily back onto that same location in the attention field. Upon the release from inhibition from the CoS node, the attention field selects a new salient location for activation, that is not typically the same as the previously examined location.

This form of visual exploration runs continuously and completely autonomously, in an ongoing sequence of shifts of attention. This ongoing sequence is interrupted when a cue is given from the outside, for example, by a human operator. The cue resets the attention field through a short burst of inhibition and acts as a mask to the input path from the color map, amplifying the specified color. When the attention field recovers from inhibition, it now selects a location matching the cued color. This attentional peak induces activation from working memory of the height value associated with that location, which can now be handed on to the reach and grasp module.

### 3.2. Time course of shape classification and pose estimation

With the activation of the cueing behavior, a peak in the attention field defines a window of attention, that channels input to the shape classification and pose estimation portion of the architecture (Figure 4). The CoS node of the cueing behavior provides a boost to the resting level boost of all estimation fields, which gets the estimation process started. The classification nodes are all equal and at resting level.

**Figure 6:**
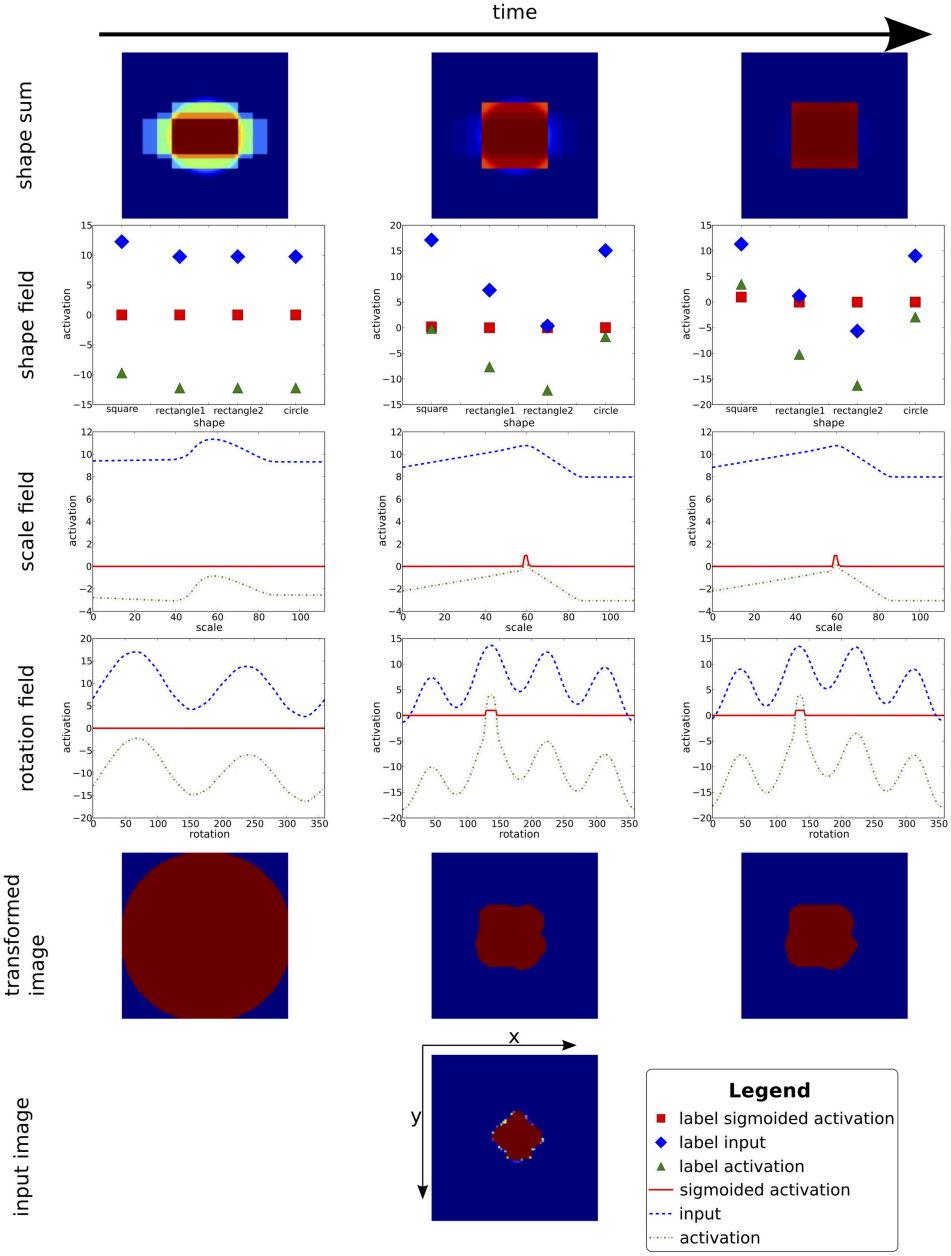
**This figure shows the time course of the convergence process of the pose estimation and shape classification processes**. In each column, the current weighted sum of shapes is shown on top. Below, inputs and activation levels of the fields representing the shape weights, as well as scale and rotation estimates are displayed (the translation estimate is not shown since the attentional blob sufficiently centers the input image, trivializing this estimation). At the bottom, the raw and transformed input image is shown. At the beginning (time passes from left to right) the transformed input is blurred out and the estimation fields only contain sub-threshold activity. While the process converges, the estimation fields select pose candidates. With the fixed pose, the shape field converges onto a classification of the base shape. Note that this is a recurrent process, that is, pose estimates and shape classification converge in parallel and support each other.

At the beginning of the process (see left column of Figure 6), the sum of shape templates in the top-down path is a homogeneous mixture of every known shape. Since all shapes are stored in a centered fashion, even this sum provide a cue to translation estimates. Over time, the pose estimation fields build up peaks, which compete within the fields for selection. As these estimates sharpen, the cross-correlations at every stage of pose transformation produce increasingly precise input to the pose fields. The match between the transformed input image and the stored shapes improves at the same time (middle column of Figure 6). The pose estimates converge somewhat earlier than the neural nodes that make shape selection, which operate on a slightly slower time scale (right column of Figure 6). At this point both the top-down pathway as well as the bottom-up pathway are fully converged onto candidate estimates, but are still reactive to changes in the input (e.g., caused by rotating or shifting the target object). Both bottom-up and top-down pathways participate in this bootstrap process.

### 3.3. Time course of movement generation

**Figure 7:**
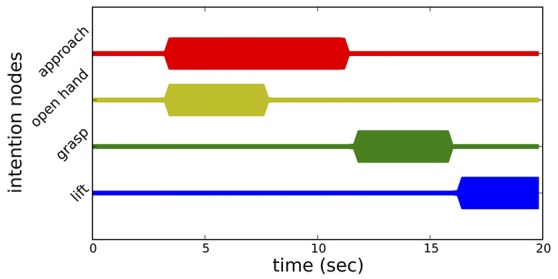
**Time course of the elementary processing units of movement generation**. Each line represents the activation level of the intention node of a behavior through line thickness.

**Figure 8:**
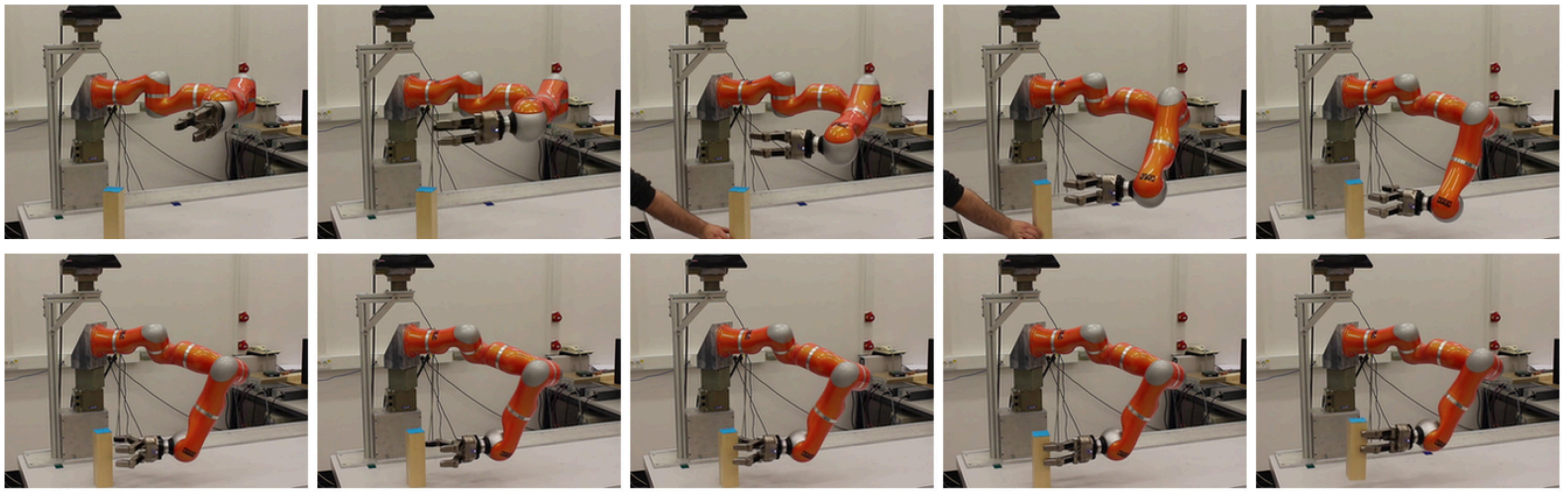
**This figure shows snapshots of a reaching and grasping trial**. The third and fourth snapshot show a human intervening in the scene by moving and rotating the target object. Shortly after this intervention, the grasp approach adapts to the new pose leading to a successful grasp in the new pose followed by lifting up of the object.

Figure 7 illustrates the time line of the neural dynamics of behavioral organization of movement generation. Initially, none of the movement intention nodes is active, since no object has been recognized yet. When all fields of the pose estimation system have stabilized a peak, movement generation is initiated. The *approach* behavior and the *open hand* behavior become active at the same time and unfold in parallel. The *open hand* behavior terminates once the hand is open, while the *approach* behavior continues until the wrist of the arm has reached a certain target point and the hand is oriented correctly. The successful completion of either behavior is signaled through the respective CoS node. Once both CoS nodes become activated, the *grasp* behavior is activated. The arm moves the remaining distance to the object while the hand is closing. Pressure sensors in the fingers signal to the CoS node of the *grasp* behavior which is activated once a grasp is detected. The *grasp* intention node is deactivated by its CoS, and the *lift* behavior is activated. The series of snapshots of the robot arm during the reaching toward and grasping of an object is shown in Figure 8. This instance of reaching and grasping contains online updating as the object is moved and rotated by the experimenter after the movement has been initiated. We examine online updating next.

### 3.4. Three experiments to probe online updating

The task is to successfully reach for and grasp an object that is positioned on the table in front of the robot and then lift it up without losing grip, even if the object's pose is changed after the beginning of a trial. To assess performance, we count a grasp and lift as successful, if the object is lifted without losing grip. Failures include tipping over the object, closing the fingers without grasping, and not lifting the object. In addition, we also count trials as failed if the experimenters have to intervene with a safety stop due to singular arm configurations or any form of collision. In some cases, the grasp was executed successfully even without precise estimates (e.g., orientation estimate is off, base shape is not detected correctly). We count such trials as errors in classification.

**Figure 9:**
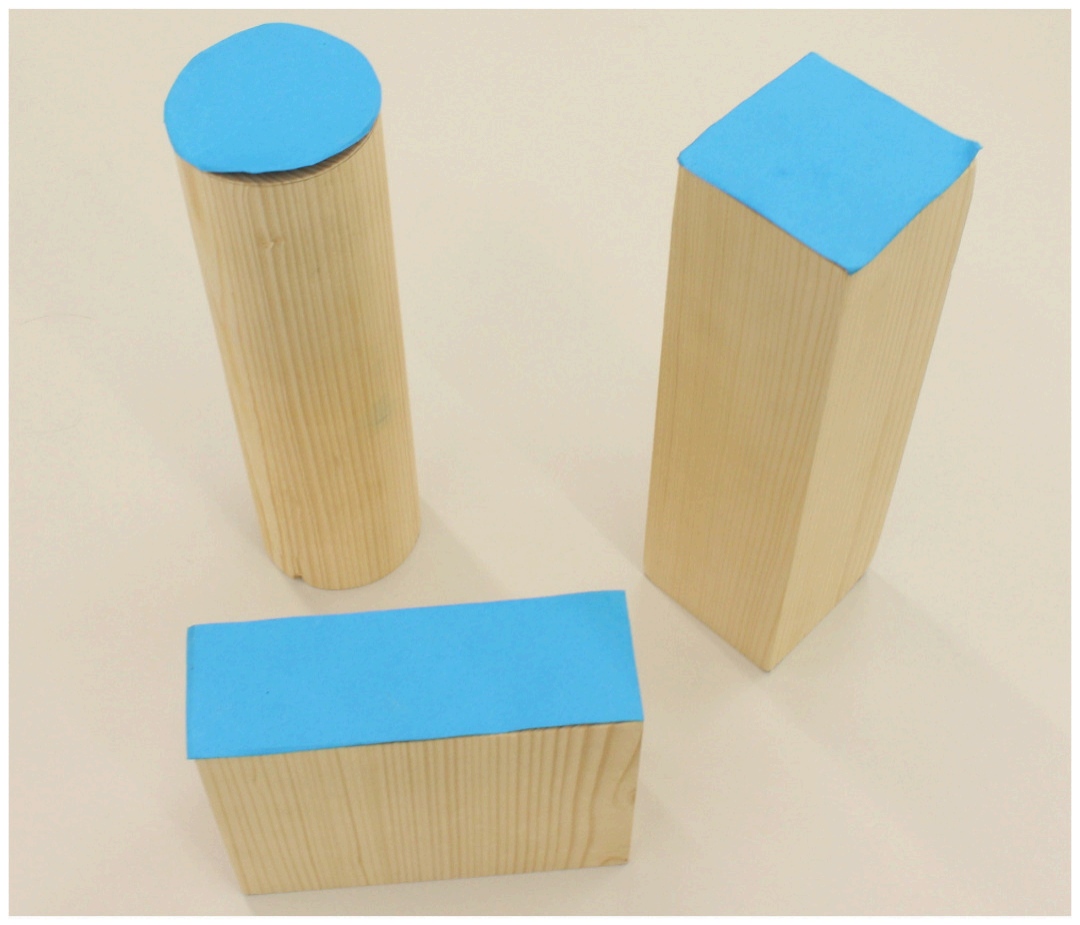
**The three simple wooden objects used in the experiments are shown**. Each object is colored blue on its top surface. Blue was used as query cue to indicate the target object.

For the experiments, we used a set of three simple wooden objects. The objects relate to the different grasps that the architecture is capable of executing: One cylinder and two cuboids, one with square base shape, the other with an oblong base shape (see Figure 9 and Table 1). The object recognition system uses three geometric shapes that loosely fit the base shapes of the objects, that is, scale and aspect-ratio of the templates are close to those of the objects.

**Table 1:** **Object sizes**.

**Object**	**Size axis 1**	**Size axis 2**	**Height**
Cylinder (cm)	7	7	25
Square (cm)	7	7	25
Rectangle (cm)	15	5	12

For practical reasons, the trunk degree of freedom of the robot was kept constant at 0° or −45° during all trials. This is a small number of trials to singular arm configurations, which our approach did not explicitly avoid. This limitation should be overcome in future implementations and illustrates how the trunk degree of freedom helps to cover a large workspace.

#### 3.4.1. Grasping without online updating

**Figure 10:**
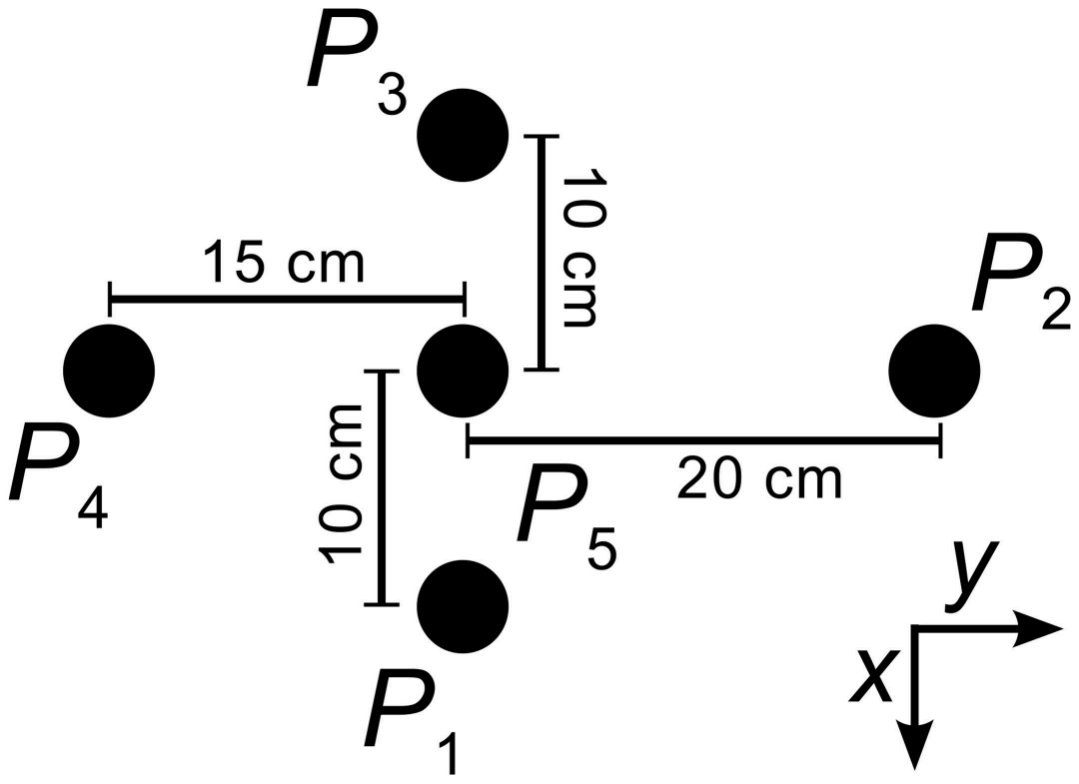
**This is the object placement layout for all experiments**. Point *P*_5_ is located at a distance of 45 cm along the *x*-axis of the world coordinate frame originating in the center of the robot's trunk. The shifts of points *P*_1_ to *P*_4_ are illustrated.

In a first experiment, we placed a single object from the object pool onto the table in front of the robot. We picked five different positions, *P*_1_–*P*_5_ (see Figure 10) and multiple orientations for the square cuboid (0°, 30°, 60°) and the oblong cuboid (0°, 45°, 90°, 135°). For the cylinder, we repeated each trial three times, for a total of 50 trials in experiment 1.

The performance of plain grasps without online updating is shown in Table 2. To minimize singular arm configurations, the sideways grasps were executed with the trunk joint at −45°, while the top grasps were executed with a trunk joint angle of 0°. Of the 50 trials, 46 were successful (92% success rate). Individual trials failed due to a singularity in the arm configuration (twice) or failed recovery from a lost peak in the estimation architecture (twice). Table 2 also contains the classification rate in all successful trials. In three trials, the base shape of the object was not detected correctly, but nonetheless the object was grasped successfully.

**Table 2:** **Results of first experiment**.

	**Cylinder**	**Square cuboid**	**Oblong cuboid**	**Total**
No. of successful trials	14	13	19	46
Success rate (%)	93.33	86.67	95	92
Successful classifications	12	12	19	43
Classification rate (%)	85.71	92.31	100	93.48

#### 3.4.2. Online updating of position

The second experiment investigates the tracking capabilities for position changes. For this experiment, we placed the cylindrical object in one of the five starting positions *P*_1_–*P*_5_. For positions *P*_1_–*P*_4_, we moved the object by hand toward position *P*_5_ once the arms started moving, covering a distance of 10 cm in roughly one to two seconds. When the object started in position *P*_5_, we instead moved the object in the direction of one of the four other starting positions by 10 cm. These eight conditions are tested three times for a total of 24 trials, which the robot performed at a fixed trunk joint angle of 0°.

Of the 24 trials, 21 were successful (87.5% success rate). In seven trials the cylinder was erroneously recognized as a square cuboid from the start or after the hand of the experimenter had touched the object to move it to the new position (see Table 3 for a listing of successful trials per condition). Three trials were counted as failed due to a safety stop of the experiment. In two of these cases, the arm configuration reached a singularity, in the third case the fingers almost collided with the object due to an erroneous position estimate. Table 3 also lists the rate of correct classification in successful trials.

**Table 3:** **Results of second experiment**.

	***P*_1_ → *P*_5_**	***P*_2_ → *P*_5_**	***P*_3_ → *P*_5_**	***P*_4_ → *P*_5_**	**Total**
No. of successful trials	3	3	3	2	11
Success rate (%)	100	100	100	66.67	91.67
Classification rate (%)	66.67	0	66.67	50	45.45
	***P*_5_** → ***P*_1_**	***P*_5_** → ***P*_2_**	***P*_5_** → ***P*_3_**	***P*_5_** → ***P*_4_**	**Total**
No. of successful trials	3	1	3	3	10
Success rate (%)	100	33.33	100	100	83.33
Classification rate (%)	100	100	100	66.67	90

#### 3.4.3. Online updating of orientation

For the third experiment, we picked the two cuboids, which require a distinct hand orientation for grasping. Each object was placed in one of the starting positions *P*_1_–*P*_5_. Once the arm started moving, we turned the object in place around 45° within about one second. We repeated this three times for each object and starting position, altering the starting orientation and turning direction, ending up with 30 trials. Grasps of the square cuboid were performed with a trunk joint angle of 45°, while the top grasp object was grasped with the trunk being at 0° (see first experiment).

Out of 30 trials, 25 were successful (83.34% success rate, see Table 4). We repeated two trials for the square cuboid due to an erroneous estimate of base shape (circle instead of square). Of the two failed trials for the square object, one was a safety stop near a singular arm configuration, while the other failed due to an error in behavioral organization (the fingers did not open). The three failed trials of the longish cuboid comprise two wrongly estimated orientations (and safety stops before collision) and one approach was aborted by the behavioral organization caused by a reverse detection in an estimation field.

**Table 4:** **Results of third experiment**.

**Square**	***P*_1_**	***P*_2_**	***P*_3_**	***P*_4_**	***P*_5_**	**Total**
No. of successful trials	2	3	3	3	2	13
Success rate (%)	66.67	100	100	100	66.67	86.67
**Oblong**	***P*_1_**	***P*_2_**	***P*_3_**	***P*_4_**	***P*_5_**	**Total**
No. of successful trials	3	1	2	3	3	12
Success rate (%)	100	33.33	66.67	100	66.67	80

## 4. Conclusion

The neural dynamics architecture presented in this paper integrates modules that have previously been developed for scene representation, concurrent object classification and pose estimation, behavioral organization, and movement generation into one big dynamical systems. Sequences of perceptual events induce reach and grasp actions, as the architecture goes through controlled instabilities. As a result, the system is open to time-varying sensor information at all times. We demonstrated on-line updating of reaching and grasping movements to shifts and rotations of the object. The architecture also responds flexibly at the level of organization. When a target object is removed, the perceptual and motor actions are abandoned and the system returns to scene exploration. When the concurrent object classification and pose estimation fail to converge, for instance, because the object is too different from a learned template, then the perceptual process terminates and the system similarly returns to scene exploration.

The stability of all relevant states in the neural dynamics is critical for both integration and online updating. Attractor states are robust to the changes in the dynamics of a component that occur as the component is coupled into the larger architecture. Instabilities, at which attractor states disappear, are controlled through the mechanism of a condition of satisfaction.

Although we have evaluated the implementation of the architecture quantitatively, the current model is a demonstration of principle, that has not yet fully exploited all features of the approach. We did not use the size estimates obtained from the object classification system, for instance, and have made use of only a small number of shape templates, which in addition resemble the target objects and do not show generalization to objects of different shape. The avoidance of obstacles was not a focus of this work. We believe that the human-like organization and the smooth temporal structure of behavior in the neural dynamics architecture will prove most useful when cognitive robots cooperate with humans. On-line updating is critical there, as human users will not always wait for their turn.

Finally, we did not yet address the issue of learning to grasp. There are two obvious parts of the architecture that could benefit from learning. One is the set of geometric shapes used during classification and pose estimation, the other is the grasp type associated with each geometric body. Naturally, the set of geometric shapes should arise from exposure to a large amount of graspable objects. Any learning process has to address the challenge of making the decision if the shape of an object can be sufficiently matched by an existing shape from the set or if the object shape should be added to the set of templates. This may also include a pruning process to remove shapes if they become obsolete by adding better-fitting shapes to the set. The links between grasp types and geometric shapes will also have to be established by a learning process. To decide if a grasp type is suitable for a geometric body (considering the base shape and the height), one may use a reinforcement learning approach by trying different grasp types for the same object and using the CoS activation (or its absence) of the grasping and lifting behaviors as positive or negative reinforcement signals. The links may also be established by learning from demonstration (see, for example, Herzog et al., 2012). If both learning of geometric shapes and links to grasp types are done concurrently, one might run into a chicken-egg problem of not being able to learn one without a mature state of the other. A developmental process of first restricting possible shapes and executable grasps to small and primitive sets and bootstrapping the architecture with increasing complexity over time is a possible procedure to overcome this dilemma.

## Author contributions

All authors designed the study. GK, SZ, and HR defined the model. GK implemented the model and performed the stimulations and experiments. GS wrote the paper, using input from the other authors.

## Funding

We gratefully acknowledge funding through the EU project “NeuralDynamics” (GS, coordinator).

### Conflict of interest statement

The authors declare that the research was conducted in the absence of any commercial or financial relationships that could be construed as a potential conflict of interest.
